# BAFF/APRIL system in pediatric OMS: relation to severity, neuroinflammation, and immunotherapy

**DOI:** 10.1186/1742-2094-10-10

**Published:** 2013-01-16

**Authors:** Michael R Pranzatelli, Elizabeth D Tate, Nathan R McGee, Anna L Travelstead, Jerry A Colliver, Jayne M Ness, Richard M Ransohoff

**Affiliations:** 1 Department of Neurology, National Pediatric Myoclonus Center and Neuroimmunology Laboratory, and Southern Illinois University School of Medicine, PO Box 19643, Springfield, IL 62794-9643, USA; 2 Flow Cytometry Center, Southern Illinois University School of Medicine, PO Box 19626, Springfield, IL, 62794-9626, USA; 3 Statistics and Research Consulting, Southern Illinois University School of Medicine, PO Box 19623, Springfield, IL, 62794-9623, USA; 4 Department of Pediatrics, University of Alabama at Birmingham School of Medicine, Birmingham, AL, 35233, USA; 5 Cleveland Clinic Foundation, The Lerner Research Institute, Mellen Center for MS Treatment and Research, Mail Code NC30, 9500 Euclid Avenue, Cleveland, OH, 44195, USA

**Keywords:** BAFF-R, Cerebrospinal fluid cytokines, Childhood neuroinflammatory disorders, Corticosteroids, Corticotropin, IVIg, Neuroblastoma, Opsoclonus-myoclonus, Paraneoplastic syndrome, Rituximab

## Abstract

**Background:**

B-cell dysregulation has been implicated but not fully characterized in pediatric opsoclonus-myoclonus syndrome (OMS), a neuroblastoma-associated neuroinflammatory disorder.

**Objective:**

To assess the role of B-cell activating factor (BAFF) and a proliferation-inducing ligand (APRIL), two critical B cell-modulating cytokines, as potential biomarkers of disease activity and treatment biomarkers in OMS.

**Methods:**

Soluble BAFF and APRIL were measured in cerebrospinal fluid (CSF) and serum by ELISA in 433 children (296 OMS, 109 controls, 28 other inflammatory neurological disorders (OIND)). BAFF-R receptors on circulating CD19+ B cells were measured by flow cytometry. A blinded scorer rated motor severity on the OMS Evaluation Scale. Immunotherapies were evaluated cross-sectionally and longitudinally.

**Results:**

The mean CSF BAFF concentration, which was elevated in untreated OMS and OIND, correlated with OMS severity category (*P* = 0.006), and reduction by adrenocorticotropic hormone or corticotropin (ACTH) (−61%) or corticosteroids (−38%) was seen at each level of severity. In contrast, CSF APRIL was normal in OMS and OIND and unaffected by immunotherapy. When the entire OMS dataset was dichotomized into ‘high’ *versus* ‘normal’ CSF BAFF concentration, the phenotype of the high group included greater motor severity and number of CSF oligoclonal bands, and a higher concentration of inflammatory chemokines CXCL13 and CXCL10 in CSF and CXCL9 and CCL21 in serum. Serum APRIL was 6.7-fold higher in the intravenous immunoglobulins (IVIg) group, whereas serum BAFF was 2.6-fold higher in the rituximab group. The frequency of B cell BAFF-R expression was similar in untreated and treated OMS. Longitudinal studies of CSF BAFF revealed a significant decline in ACTH-treated patients (with or without rituximab) (*P* < 0.0001). Longitudinal studies of serum APRIL showed a 2.9-fold increase after 1 to 2 g/kg IVIg monotherapy (*P* = 0.0003).

**Conclusions:**

Striking distinctions in BAFF/APRIL signaling were found. OMS displayed heterogeneity in CSF BAFF expression, which met many but not all criteria as a potential biomarker of disease activity. We speculate that CSF BAFF may have more utility in a biomarker panel than as a stand-alone biomarker, and that the selective upregulation of both serum APRIL by IVIg and BAFF by rituximab, as well as downregulation of CSF BAFF by ACTH/steroids, may have utility as treatment biomarkers.

## Introduction

Opsoclonus-myoclonus syndrome (OMS) is a neuroinflammatory disorder of children and adults, which is demonstrably paraneoplastic in about 50% of the cases [1]. Our research on CSF in pediatric OMS has shown that B cells are expanded [2], B cell chemoattractant CXCL13 [3] and B/T cell chemoattractant CXCL10 [4] are likewise overexpressed, and 35% of the patients harbor oligoclonal bands. [5] Responses to current immunotherapeutic strategies, such as intravenous immunoglobulins (IVIg), corticosteroids, and anti-CD20, also are consistent with B-cell involvement [2].

Two critical cytokines for B-cell activation, proliferation, homeostasis, and survival are a proliferating-inducing ligand (APRIL) and B-cell activating factor (BAFF) [6]. They are members 13 and 13B, respectively, of the tumor necrosis factor ligand superfamily (TNFLS) [7,8]. Both are expressed by astrocytes [9,10], and also dendritic cells, macrophages, and monocytes [11]. In human cerebrospinal fluid (CSF) and serum, they are found in soluble form and often studied together in neuroinflammatory diseases [12-14]. The BAFF receptor (BAFF-R or BR3), one of three receptors to which BAFF binds [11], is the principal BAFF receptor [15] expressed by nearly all mature B cells [10].

Our preliminary work on BAFF showed an increase in OMS and decrease by corticotropin (ACTH) or corticosteroids [16]. The present report in an increased OMS sample size tests the hypothesis that BAFF is a biomarker of disease activity and treatment biomarker (not diagnostic biomarker). It includes additional immunotherapy cross-sectional groups; analysis of the relation to OMS severity (video documented and scored) and duration; comparison with APRIL, inflammatory chemokines, oligoclonal bands, and lymphocyte subsets; longitudinal studies of BAFF and APRIL, and comparative studies in other inflammatory neurological disorders (OIND). To identify the ‘phenotype’ of the patients with high CSF values compared to those with normal (control-like) values, we are now able to dichotomize the dataset. We also measured the BAFF-R receptor on circulating B cells in the current study.

## Methods

### Patients and procedures

This prospective, case–control, translational research study is part of the ‘Study of Cytokines in Children with Opsoclonus-Myoclonus Syndrome’ (ClinicalTrials.gov NCT 00806182) [3]. Approximately 300 children were recruited through the National Pediatric Myoclonus Center, regardless of severity or treatment status. Inclusion criteria included clinically confirmed diagnosis of OMS and ages 0.6 to 18 years, without restriction due to gender, race, ethnicity, or socioeconomic status. Exclusion criteria were contraindications to lumbar puncture or sedation/anesthesia, drug treatments outside the study scope, or concurrent autoimmune disorders.

The cross-sectional part of the study involved 276 children with OMS, mean age (SD) 3.8 ± 3.0 years (range 0.89 to 17 years; boys *n *= 120; girls *n *= 156), who were enrolled after consent was signed by parents and assent by older children. OMS groups included untreated, currently treated, and previously treated patients. Videotapes were made using a standardized procedure and later scored blinded on the OMS Evaluation Scale to yield a total score [2]. Further details of treatment and scoring are provided in the figure legends. The children underwent a morning lumbar puncture following our specified protocol [2].

Controls comprised children with various non-inflammatory neurological disorders (NIND), including ataxia, developmental disorders, headache, movement disorders, seizures, and miscellaneous disorders. Their mean age was 8.4 ± 5.6 years, and 96 were boys and 91 were girls. Children with OIND, who were evaluated locally and through the Center for Pediatric-Onset Demyelinating Disease in Alabama, served as a comparison group for specificity. Their diagnoses included acute disseminated encephalomyelitis, encephalitis, meningitis, multiple sclerosis and related disorders, and miscellaneous disorders. The OIND mean age was 7.7 ± 5.8 years (range 0.1 to 18 years; boys *n *= 17; girls *n *=23).

Two longitudinal OMS studies were performed. In the BAFF study, 42 children from the cross-sectional study were treated using ACTH-based conventional therapy with (*n* = 31) or without (*n* = 11) rituximab. There were 16 boys and 26 girls, mean age 3.7 ± 2.8 years (range 1.8 to 18 years). CSF was obtained before and at 8.0 ± 2.1 months of treatment. In the APRIL study, 20 new children with OMS were recruited for monotherapy with intravenous immunoglobulins (IVIg) under FDA BB-IND No. 5839. There were 11 boys and nine girls, mean age 4.6 ± 1.4 years (range 1.8 to 6.4 years). They received standard monthly clinical doses of 1 or 2 g/kg, and serum was collected before and at 4.1 ± 3.9 months of treatment.

### Cytokine/chemokine detection

CSF and serum were collected on ice, aliquotted, and stored at −80°C in biorepository freezers. Samples were thawed on the assay day and BAFF and chemokines were measured using Quantikine ELISA kits per instructions by the manufacturer (R&D Systems, Inc., Minneapolis, MN, USA). The comparison chemokine panel included CXCL9, CXCL10, CXCL12, CXCL13, CCL17, CCL21, and CCL22 kits from the same vendor. Human APRIL ELISA kits were purchased from eBioscience (formerly Bender MedSystems, Vienna, Austria). Assays were performed in duplicate on undiluted samples, each 96-well plate containing both control and OMS samples. Samples with values above the highest standard were re-run at a 1:2 or higher dilution. Outliers were verified by repeat measurement. The kit user had no patient contact. BAFF sensitivity was 0.73 ± 6.7 pg/mL in CSF, and 1.5 to 11.9 pg/mL in serum; APRIL, 0.4 pg/mL. The inter-assay coefficient of variance (CV) was 9.4% (*n* = 22) for CSF BAFF, 8.5% (*n* = 20) for serum BAFF, 7.8% (*n* = 10) for CSF APRIL, and 4.8% (*n* = 8) for serum APRIL. The corresponding intra-assay CV was 4.8% (*n* = 11), 6.0% (*n* = 10), 7.2% (*n* = 6), and 6.7% (*n* = 6). Freezer storage time and the concentration of CSF BAFF (*P* = 0.21) or APRIL (*P* = 0.16), or serum concentrations, were not correlated.

### BAFF-R and lymphocyte subsets

BAFF-R receptors were measured *ex vivo* by flow cytometry [17]. Peripheral venous blood was delivered to the flow cytometrist within 1 h of collection. A 100 μL aliquot was blocked with 0.2 mg/mL normal mouse IgG for 15 min at room temperature. Directly conjugated monoclonal antibody probes (anti-BAFF-R, anti-CD19, anti-CD45), purchased from Becton Dickinson (San Jose, CA, USA), were added to the remaining cell suspension. Then 2 mL FACS lysing solution was added to lyse erythrocytes, followed by a 10-min incubation at room temperature in the dark. The assay suspension was centrifuged at 4°C for 7 min at 600 × g, supernatant was removed, the pellet was washed twice with 2 mL cold FACS buffer, and recentrifuged. After decanting, 100 μL of 1% paraformaldehyde was added, and after 5 min at room temperature in the dark, there was another centrifugation, decanting, and resuspension of cells in FACS buffer.

Lymphocyte subsets, measured by flow cytometry as described previously [2], included cells that were CD19+CD3- (B cells), CD4+CD3+ (T helper/inducer cells), CD8+CD3+ (T cytotoxic/suppressor cells), TCRγδ+ (gamma/delta T cells), or CD16/56+ (NK cells). Data were acquired on a FACSCalibur cytometer and analyzed using Cell Quest (Becton-Dickinson, San Jose, CA, USA). Quality control measures were rigorous, as reported elsewhere [2]. The flow cytometrist had no patient contact.

### Statistical analysis

Because of inter-individual differences and the large range of values inherent in human cytokine and chemokine data, both means and medians were analyzed. Cross-sectional groups were compared by analysis of variance (ANOVA) with Tukey post-hoc tests, and primary comparisons were between controls and untreated OMS, and between untreated and treated OMS. Medians were analyzed secondarily by the Kruskal-Wallis test with Dunn’s post-hoc test. Two group comparisons were made by two-tailed *t* tests or Mann–Whitney tests, depending on variance, but pre- and post-treatment comparisons utilized the paired *t* test. Percentages were compared by Fisher’s exact test. Correlation analysis utilized Pearson correlations. Although the mean age of the OMS group was significantly lower than in controls or the OIND group (which did not differ), no correlation between age of controls and CSF BAFF concentration (*P* = 0.72) or CSF APRIL (*P* = 0.19) was found, and the age range of the groups was equivalent.

Secondary analysis was performed on the OIND group compared to the other groups. Also, OMS immunotherapy groups were bundled based on common treatment effects for graphic presentation. Lastly, the dataset was dichotomized based on cytokine levels 2 SD above the control mean to determine if two different phenotypes emerged, and family-wise Bonferroni corrections (α at 0.5/n) were performed separately on six clinical variables (*P* < 0.008), nine chemokines/cytokines and oligoclonal bands (*P*  < 0.0055), and five lymphocyte subsets (*P*  <  0.01) to control for the multiple comparisons that were made.

## Results

### BAFF cross-sectional study

**Figure 1:**
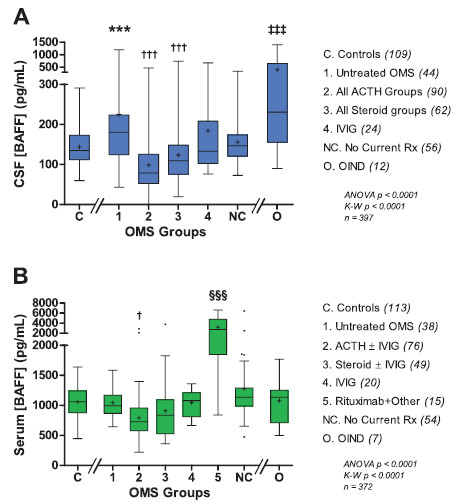
**Cross-sectional BAFF concentrations. **(**A**) CSF BAFF. Box and whisker graphs depict the mean as a plus sign, the median as a line within the box, interquartile ranges as the upper and lower box lines, and the range as Tukey error bars. Primary statistical comparisons were of means for untreated OMS *versus* controls (*) and treated *versus *untreated OMS (†). Statistical significance level is indicated by the number of symbols: * 0.05 > *P* ≥0.01, *** *P *≤0.0001. The pre-bundled treatment groups were ACTH (*n *= 37), steroids (*n* = 22), IVIG (*n *= 24), ACTH + IVIg (*n *= 39), steroids + IVIg (*n *= 27), ACTH + other (rituximab, chemotherapy, or steroid sparers) (*n *= 14), and steroids + other (*n *= 13). None of the three ACTH groups differed from each other in BAFF concentration, so they were bundled into ‘All ACTH Groups’. Steroid groups were bundled the same way. OIND was significantly different than all other groups (‡). The mean ACTH dose in the combined ACTH groups was 28 ± 22 IU/m^2^/day (alternate day doses averaged to provide a daily dose equivalent). The mean steroid dose was 1.3 ± 1.3 mg/kg/day. (**B**) Serum BAFF. The ACTH monotherapy group and the ACTH + IVIg group, which were not significantly different in BAFF concentrations, were bundled into group 2; steroids were handled likewise in group 3. The ‘Rituximab + Other’ group included rituximab, IVIg, and either steroids or ACTH.

Differences in CSF BAFF were highly significant between groups (Figure 1A). Mean CSF BAFF was 57% higher in untreated OMS than in controls. Twenty-three percent of untreated OMS had CSF BAFF concentrations >2 SD above the control mean. The three ACTH groups are shown bundled, as are the three steroid groups, for lack of significant difference in CSF BAFF among them. CSF BAFF was 56% lower in ‘All ACTH Groups’ and 45% lower in ‘All Steroid Groups.’

In comparisons of individual OMS treatment groups *versus* untreated OMS (data not shown), significantly lower mean CSF BAFF concentrations (pg/mL) were found for ACTH (88 ± 59, -61%, *P* < 0.0001), steroid (140 ± 148, -38%, *P* = 0.002), ACTH + IVIg (113 ± 92, -50%, *P* < 0.0001), steroid + IVIg (114 ± 42, -49%, *P* = 0.0002), ACTH + other (89 ± 76, -60%, *P* < 0.0001), and steroid + other (115 ± 52, -49%, *P* = 0.005). In each of those groups, the mean BAFF concentration was below, though not significantly different than, the control mean. Of actively treated patients, 22% had CSF BAFF concentrations below the lowest controls. In previously treated patients, mean CSF BAFF concentration did not differ significantly from controls.

The OIND group also manifested an elevated mean CSF BAFF concentration compared to untreated OMS (*P* = 0.04) and controls (*P* < 0.0001), though there was no significant difference in medians. In four OIND, whose diagnoses included encephalitis, neurolupus, and developmental delay, the CSF BAFF concentration was above the highest control in the 369 to 1,398 pg/mL range. The CSF:serum BAFF ratios did not differ between these groups.

ACTH and steroid monotherapy groups did not differ significantly in CSF BAFF concentrations (*P* = 0.06, *t*-test and Mann–Whitney test). When all ACTH groups (*n* = 90) were compared to all steroid groups (*n* = 62), there was no significant difference in CSF BAFF concentrations: 91 ± 78 vs 123 ± 95 pg/mL, respectively. Only the combined ACTH group was significantly lower than the IVIg group (184 ± 136 pg/mL).

The mean CSF/serum BAFF ratio was 64% higher in untreated OMS than in controls (*P* < 0.0001). Compared to untreated OMS, there were significant ratio reductions in treated groups. The lowest ratios were for ACTH (0.11 ± 0.06, -55%, *P* = 0.0007), steroids (0.12 ± 0.05, -49%, *P* = 0.02), ACTH + IVIg (0.14 ± 0.10, -38%, *P* = 0.02), steroid + IVIg (0.15 ± 0.06, -49%, *P* = 0.008), ACTH + other treatments (0.07 ± 0.05, -70%, *P* = 0.009), and steroid + other treatments (0.08 ± 0.05, -63%, *P* = 0.02).

The mean serum BAFF concentration did not reflect CSF concentrations (Figure 1B). It was significantly higher only in rituximab-treated patients receiving combination immunotherapy. The BAFF elevation was two-fold in the ACTH + other group and 2.3-fold in the steroid + other group. When the ACTH + other and steroid + other groups were combined, the 50% treated with rituximab had a 2.6-fold higher serum BAFF (3,186 ± 1,774 pg/mL) than the rest treated with chemotherapy or steroid sparers (1,236 ± 779 pg/mL) (*P* < 0.0001).

### APRIL cross-sectional study

**Figure 2:**
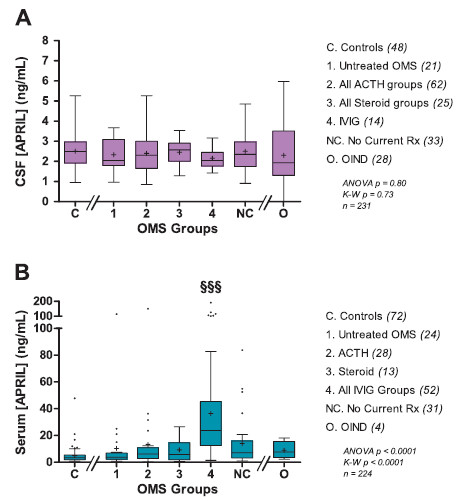
**Cross-sectional APRIL concentrations. **(**A**) CSF APRIL, expressed as ng/mL (1000 × pg/mL). The pre-bundled treatment groups were ACTH (*n *= 33), steroids (*n *= 11), IVIg (*n *= 14), ACTH + IVIg (*n *= 29), steroids + IVIg (*n* = 14). (**B**) Serum APRIL, expressed as ng/mL. The three IVIg groups (IVIg, ACTH + IVIg, steroids + IVIg) did not differ significantly from each other in serum APRIL concentration, so they were combined into ‘All IVIg groups’. Compared to controls, the serum APRIL concentration was higher by 6.7-fold in the IVIg monotherapy group (32.6 ± 34 ng/mL), by 8.2-fold in the ACTH + IVIg group (39.9 ± 45 ng/mL), and by 6.9-fold in the steroid + other group (33.3 ± 26 ng/mL). IVIg was infused monthly, and most evaluations were scheduled just before the next IVIg was due.

The CSF concentration of APRIL did not differ significantly between the control group and OMS cross-sectional groups (Figure 2A). Neither ACTH nor steroids lowered the concentration of CSF APRIL, and IVIg also had no effect. CSF APRIL was not elevated in the OIND group.

Combined IVIg treatment groups displayed significantly higher serum APRIL concentrations (Figure 2B); so did individual IVIg groups (see figure legend). In contrast, serum APRIL in ACTH- or steroid-treated groups did not differ from untreated OMS or controls, which did not differ from each other. The APRIL concentration in serum did not reflect that in CSF.

As a result of increased serum APRIL, the CSF:serum APRIL ratio was lower by 53% in the IVIg monotherapy group, 60% in the ACTH + IVIg group, and 81% in the steroid + IVIg group compared to untreated OMS. In controls, the CSF:serum APRIL ratio was 5.8-fold higher than the CSF:serum BAFF ratio (*P*  <  0.0001); in untreated OMS, it was 2.8-fold higher (*P*  <  0.0001).

### Relation to clinical data

**Figure 3:**
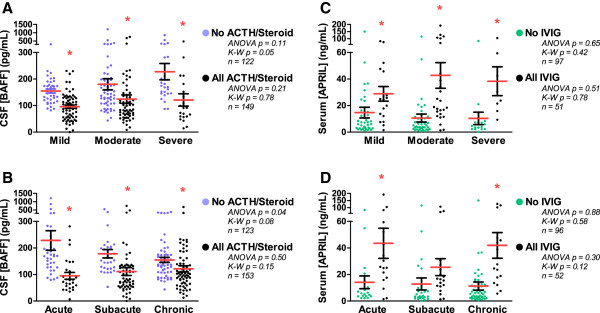
**CSF BAFF and serum APRIL concentration *versus *OMS severity and duration category. **(**A**) Dot plot comparison of BAFF *versus *OMS severity in all ACTH/steroid groups *versus *all other OMS groups. Asterisk denotes *P *< 0.05 by two-tailed *t* tests within categories. Severity categories were pre-defined based on total score as mild (0–12), moderate (13–24), or severe (25–36). Between-category comparisons were made by ANOVA and Kruskal-Wallis tests. (**B**) Comparison of BAFF *versus *OMS duration in all ACTH/steroid groups *versus* all other OMS groups. OMS duration was pre-defined as acute (0 to 3 months), subacute (>3 months to 1 year), or chronic (>1 year). (**C**) Serum APRIL concentrations *versus *OMS severity in all IVIg groups *versus *all non-IVIg groups. (**D**) Serum APRIL *versus *OMS duration in all IVIg groups *versus *all non-IVIg groups.

Patients were separated according to OMS severity or duration. Mean CSF BAFF concentration increased significantly with OMS severity category in the combined OMS dataset (*n* = 271) (*P* = 0.006, ANOVA); so did the median (*P* = 0.02, Kruskal-Wallis test). For OMS duration category, there was no significant relation to CSF BAFF (*P* = 0.11). The CSF:serum BAFF ratio correlated with OMS total score (r = 0.38, *P* = 0.018), OMS duration (r = −0.48, *P* = 0.0018), and CSF B cell frequency (r = 0.59, *P* = 0.0087). When all groups receiving either ACTH or steroids were analyzed separately from all remaining OMS groups, they manifested the lowest CSF BAFF concentrations regardless of OMS severity (Figure 3A) or duration category (Figure 3B). Only in the remaining OMS groups did CSF BAFF concentration trend with OMS severity and OMS duration.

For serum APRIL, there were no significant differences between severity or duration categories when the entire OMS dataset was used (*P* = 0.27). However, when the OMS dataset was split into no IVIg groups *versus* all IVIg groups, significant differences were found for IVIg at each level of OMS severity (Figure 3C) and for two categories of OMS duration (Figure 3D). There was no significant difference across severity categories.

The tumor OMS group (1.14 ± 95 pg/mL, *n* = 137) and the non-tumor OMS group (1.11 ± 0.68 pg/mL, *n* = 130) did not differ significantly in mean serum BAFF concentration (*P* = 0.79), or in medians (*P* = 0.42). Mean serum APRIL concentration in the tumor group (30.3 ± 70 ng/mL) and non-tumor group (19.7 ± 30 ng/mL) also was not significantly different (*P* = 0.22).

The OMS combined dataset was analyzed for various correlations. CSF BAFF, not APRIL, correlated with CSF leukocyte count (*P* = 0.0003, Pearson correlation), though the r value was low (r = 0.24). There were no significant correlations with CSF IgG concentration or the albumin ratio.

#### Secondary analysis

**Figure 4:**
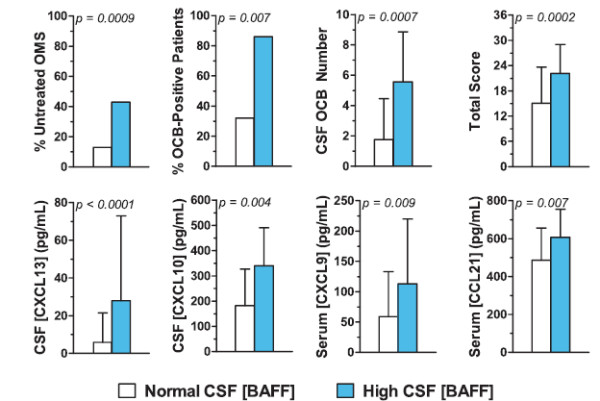
**Dichotomization of CSF BAFF concentration into ‘high’ and ‘normal’ groups for phenotyping. **Percentages were compared by Fisher’s exact tests and means by *t *tests. The high and normal groups did not differ significantly in gender ratio, patient age, or OMS duration (data not shown).

The whole OMS dataset was divided into two groups as described under statistical analysis (Figure 4). In the resulting ‘high’ BAFF group (*n* = 23), there was a significantly higher concentration of CSF CXCL13 and CXCL10, CSF oligoclonal band number and frequency, and serum CXCL9 and CCL21 compared to the ‘normal’ BAFF group (*n* = 253). The clinical characteristics of this group were greater total score (motor severity) and a higher frequency of untreated patients. CSF T cell frequency was lower in the ‘high’ BAFF group (80.3 ± 8.1%) than the ‘normal’ BAFF group (85.2 ± 7.0%) (*P* = 0.004). There were no significant between-group differences after Bonferroni corrections for CSF or serum APRIL, plasma CXCL12, serum CCL17, serum CCL22, or for CSF lymphocyte subset frequencies (CD19+ B cells, γδ T cells, CD4+ T cells, CD8+ T cells, or NK cells) (data not shown).

Because certain treatments alter CSF BAFF concentrations, the analysis was then performed only for untreated OMS, for which there were 10 ‘high’ and 34 ‘normal,’ with non-significant trends: CSF CXCL13 (*P* = 0.06), serum CXCL9 (*P* = 0.08). When the sample was increased by inclusion of all non-ACTH/steroid groups, there were 10 ‘high’ and 106 ‘normal’. The statistically significant results were for CSF CXCL13 (*P* = 0.01), CSF OCB frequency (*P* = 0.04) and number (*P* = 0.03), serum CXCL9 (*P* = 0.02), and total score (*P* = 0.02); CXCL10 and CCL21 were not significant. Thus, the ‘high’ group appeared statistically underpowered when the dataset was delimited.

### BAFF-R

BAFF-R was expressed on almost all circulating CD19+ B cells, whether from controls or OMS. Mean BAFF-R frequency was 94.5 ± 9.3% for untreated OMS (*n* = 6), 97.1 ± 5.4% for conventionally-treated OMS (*n* = 9), 90.1 ± 12.2% for multimodal immunotherapy (*n* = 13), and 96.9 ± 2.2% in controls, with no significant differences between groups (*P* = 0.45). Median frequencies also did not differ significantly (*P* = 0.62, Kruskal-Wallis test).

### Longitudinal CSF BAFF study

The mean CSF BAFF concentration in pre-treatment OMS was higher than in controls (*P* < 0.0001). It declined significantly in children receiving ACTH-based immunotherapy (Figure 4A), whether or not they also received rituximab (Figure 4B,C). Of the 42 patients, BAFF decreased in 36, did not change in two, and increased in four. The only distinguishing characteristic of patients showing a BAFF decrease was lower CSF CXCL13 (2.6 pg/mL) than the others (4.1 pg/mL) (*P* = 0.02). Post-treatment CSF BAFF concentration was in the normal range. Although there was an overall 71% reduction in motor severity (total score) (*P* < 0.0001), the change in BAFF did not correlate with the change in total score, even when patients without a decrease in BAFF were excluded (r = 0.19, *P* = 0.29).

### Longitudinal serum APRIL study

**Figure 5:**
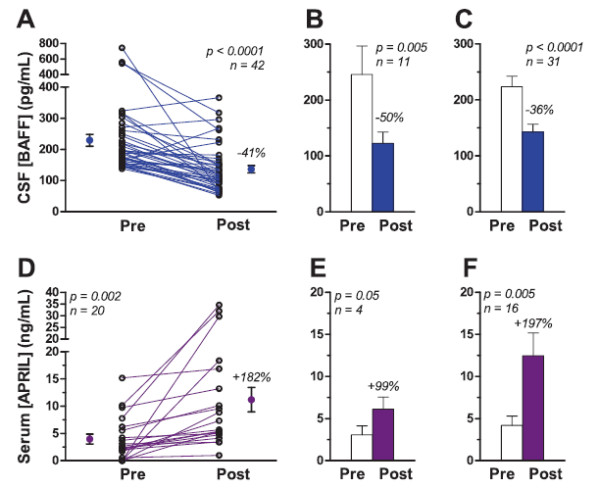
**Longitudinal effects of immunotherapy on CSF BAFF and serum APRIL. **(**A**) CSF BAFF pre- and post-treatment with ACTH-based combination immunotherapy. Group means with SEM and percent reduction are shown to either side of the line plot. (**B**) ACTH-based immunotherapy without rituximab. Comparisons were made by paired *t *tests. (**C**) ACTH-based immunotherapy with rituximab. Pre-treatment means in Figures B and C did not differ significantly; neither did post-treatment means. (**D**) Pre- and post-IVIg treatment serum APRIL. (**E**) Data from 1 g/kg IVIg dose. (**F**) Data from 2 g/kg IVIg dose. No significant differences were found between pre-treatment means or between post-treatment means in Figures E and F.

The post-IVIg serum APRIL concentration (Figure 5D) was significantly higher than the pre-treatment concentration (mean by 2.8-fold, median by 2.6-fold). Serum APRIL was increased in all patients, though the amount of change varied greatly between patients. In six patients, the post-IVIg APRIL concentration was >1 SD above the control mean; in four, >2 SD. The increase was significant in children who received 2 g/kg (Figure 5F), but the sample size was small in the 1 g/kg group (Figure 5E).

## Discussion

This study provides new insights on the BAFF/APRIL system in pediatric OMS. Correlation of CSF BAFF with clinical severity and co-segregation of high CSF BAFF with CSF inflammatory chemokines and oligoclonal bands suggest a potential role of CSF BAFF as one of several biomarkers of disease activity in OMS. BAFF also showed promise as a treatment biomarker in its remarkable sensitivity to ACTH or corticosteroids in cross-sectional as well as longitudinal studies. APRIL was not a biomarker of disease activity in OMS, but showed a striking treatment effect of IVIg. BAFF-R expression on circulating B cells was not altered in OMS, nor is it in multiple sclerosis [10] or myasthenia gravis [18].

The fact that CSF BAFF and APRIL did not trend in the same direction in OMS is not without precedent [12]. Although both are increased in neuropsychiatric lupus [12,14], only CSF BAFF is increased in untreated multiple sclerosis [19,20]. Another difference between BAFF and APRIL signaling in neuroinflammatory diseases is that both CSF and serum BAFF are elevated in neuro-Behcet’s disease [13], whereas only CSF BAFF is elevated in multiple sclerosis. The highest CSF BAFF concentrations in multiple sclerosis were found in patients with more than six oligoclonal bands [21], which is similar to the higher band counts we found in OMS. Serum BAFF and APRIL have not reflected CSF levels in other disorders [9,13,19] or in the present study.

Differences in APRIL and BAFF responses also may reflect differences in their functions [11]. Both BAFF and APRIL bind to BCMA and TACI receptors, however, only BAFF binds to BAFF-R, and APRIL also binds to surface proteoglycans [21]. The expression of these receptors differs on pre-immune B cells and antigen-experienced B cells (memory B cells and long-lived plasma cells) [22]. BAFF and APRIL may localize to different anatomic niches, with distinctive local interactions. APRIL modulates certain aspects of B cell activation and isotype switching [22]. Also, BAFF plays a role in T cell activation and polarization to Th1 [11,15], and APRIL suppresses Th2 cytokine production and antibody responses *in vitro*[23].

Extraordinary differential effects of immunotherapy were found for APRIL and BAFF. To our knowledge, increased serum APRIL concentration as an effect of IVIg therapy has not been reported previously in a neurological disorder. The only previous report we encountered was of 11 children with Kawasaki disease, an acute vasculitis that responds to IVIg [24], in whom an IVIg dose of 2 g/kg raised APRIL (6.7-fold) but lowered BAFF (−41%) in plasma. In OMS, we found no IVIg-lowering effect on serum BAFF. Serum BAFF increased only after rituximab therapy, which we showed to be an early response to B cell depletion in OMS [25]. Only ACTH and corticosteroids lowered CSF BAFF, but they did not affect CSF APRIL. Previously, a reduced concentration of serum BAFF has been reported in disorders with elevated BAFF, such as in Wegener’s granulomatosis [26]. These differences might be clinically exploited should they be found to serve as response predictive biomarkers (identifying subpopulation according to response potential) or response identification (relating biological and clinical responses to treatment) biomarkers in longitudinal studies.

The clinical impact of IVIg-induced elevation in serum APRIL is difficult to predict from observational data. In diseases associated with increased APRIL in serum, APRIL is thought to have a pathologic role. However, IVIg induces clinical improvement in OMS [1], and APRIL is not elevated in untreated OMS, raising the possibility that boosting serum APRIL could be therapeutic in OMS. Interestingly, APRIL has been suggested to be involved in downregulation of serological and clinical activity in patients with systemic lupus erythematosus [27]. This view is consistent with the proposed mode of IVIg action, which is thought to be immunomodulation of the cytokine network [28]. However, modulation of APRIL may not be the mechanism involved.

Treatment-induced reduction of BAFF in the central nervous system might decrease BAFF-dependent survival of plasma cells [9], which express BAFF-R [29]. In pediatric OMS, elevated CSF BAFF (not APRIL) correlated with CSF cerebellar autoantibodies [30], though the effect of treatment was not studied. Increased serum BAFF after rituximab, which may be important to B-cell repopulation [31], could also increase survival of autoreactive circulating B cells [29].

BAFF adds to the evolving picture of B-cell involvement in OMS, which includes CSF B-cell subset expansion [2], positive oligoclonal bands [5], intrathecal over-production of CXCL13 [3], selective over-expression of CXCR5 receptors on CSF memory B cells [3], and clinical response to adjunctive anti-CD20 monoclonal antibody rituximab [1]. One potential limitation of CSF BAFF as a biomarker is its inter-individual variability. Moreover, only 23% of patients with untreated OMS had CSF BAFF concentration >2 SD above the control mean. This shows significant overlap of CSF BAFF values between patients and controls. Our current research explores the hypothesis that inflammatory cytokines, none of which is elevated in all patients, may have greater predictive value in OMS as a biomarker cluster than individually.

In conclusion, BAFF, not APRIL, joins a short list of other putative CSF biomarkers of disease activity in pediatric OMS that includes B cell frequency (total B cells and B-cell subsets), B-cell chemoattractants (CXCL10, CXCL13), and oligoclonal bands [2-5]. Such molecules necessary for B-cell recruitment, activation, and survival may be working together to promote neuroinflammation [20]. Because of the exceptional sensitivity of CSF BAFF to peripherally administered ACTH or corticosteroid therapy, its potential utility as a response predictive biomarker or response identification biomarker warrants validation. The immunomodulatory effect of IVIg on APRIL signaling merits evaluation in a variety of neuroinflammatory disorders.

## Abbreviations

ACTH: Adrenocorticotropic hormone or corticotropin; APRIL: A proliferating-inducing ligand; ANOVA: Analysis of variance; BAFF: B cell activating factor; BAFF-R: BAFF receptor; CD: Cluster of differentiation; ELISA: Enzyme-linked immunoassay; IVIg: Intravenous immunoglobulins; NIND: Non-inflammatory neurological disorders; OIND:Other inflammatory neurological disorders; OMS: Opsoclonus-myoclonus syndrome.

## Competing interests

Richard M. Ransohoff is a member of the Scientific Advisory Board of Chemocentryx and holds stock in the company. He is a member of the Scientific Advisory Board of Vertex. The rest of the authors have nothing to disclose.

## Authors’ contributions

MRP was the principal investigator, and participated in the drafting/revising of the manuscript, the study concept and design, acquisition of data, analysis and interpretation of data, and statistical analysis. EDT was the co-investigator, and participated in patient recruitment and evaluation, study concept and design, acquisition of data, and revision of the manuscript. NRM was the laboratory researcher, and participated in the acquisition and analysis of data, and statistical analysis. ALT was the flow cytometrist, and participated in the acquisition and analysis of data, and revising of the manuscript. JAC was the study statistician, and participated in the review of the manuscript. JMN participated in OIND patient recruitment and evaluation, and vetting of the manuscript. RMR was the project consultant, and revised the manuscript. All authors have read and approved the final version of the manuscript.
